# Pre-Lens Tear Meniscus Height, Lipid Layer Pattern and Non-Invasive Break-Up Time Short-Term Changes with a Water Gradient Silicone Hydrogel Contact Lens

**DOI:** 10.3390/life12111710

**Published:** 2022-10-26

**Authors:** Raúl Capote-Puente, María-José Bautista-Llamas, Caterina Manzoni, José-María Sánchez-González

**Affiliations:** 1 Vision Research Group (CIVIUS), Department of Physics of Condensed Matter, Optica Area, University of Seville, 41012 Seville, Spain; 2 Department of Materials Science, Optics and Optometry Area, University of Milano-Bicocca, 20122 Milan, Italy

**Keywords:** pre-lens tear film, lipid pattern, non-invasive break-up time, contact lens

## Abstract

To evaluate pre-lens tear film volume, stability and lipid interferometry patterns with a silicone hydrogel water content contact lens, a novel, noninvasive, ocular-surface-analyzer technology was used. A prospective, longitudinal, single-center, self-control study was performed in daily or monthly replacement silicone hydrogel contact lens wearers. A tear film analysis was achieved with the Integrated Clinical Platform (ICP) Ocular Surface Analyzer (OSA) from SBM System. The subjects were reassessed, with the contact lens, after 30 min of wearing to quantify the volume, stability and lipid pattern of the short-term pre-lens tear film. Lipid layer thickness decreased from 2.05 ± 1.53 to 1.90 ± 1.73 Guillon patterns (*p* = 0.23). First pre-lens NIBUT decreased from 5.03 ± 1.04 to 4.63 ± 0.89 s (*p* = 0.01). Mean pre-lens NIBUT significantly increased from 15.19 ± 9.54 to 21.27 ± 11.97 s (*p* < 0.01). Lid opening time significantly increased from 26.36 ± 19.72 to 38.58 ± 21.78 s (*p* < 0.01). The silicone hydrogel contact lens with water gradient technology significantly increased the mean pre-lens NIBUT and lid opening time. Lehfilcon A suggested an improvement in contact lens wearers with tear film instability or decreased subjective symptoms of dry eye disease.

## 1. Introduction

In recent years, soft contact lenses (SCLs), and particularly silicone hydrogels (SHs), have experienced constant changes by the specialized industry, promoting the development of materials, designs and treatments with greater biocompatibility with human tissue, which provide better properties for corneal physiology, eye comfort and wettability [1,2,3,4]. Despite the introduction of new materials and surfactants in contact lens design [5,6], SH-SCL users continue to report dryness and eye discomfort at some time in the day [7], representing one of the main causes of leaving CLs [8]. Crucial comfort factors could be related to changes generated by interactions of the tear film within ocular tissues [9]. Although the exact etiology remains unknown, there are numerous factors related to discomfort in SH-SCL users. Some factors may be susceptible to their environment or due to multifactorial circumstances, such as mode of use, materials, wettability, fitting, tear film fluctuations, multipurpose solution composition, hygiene protocol, environmental exposure or patient lifestyle [6,10,11]. Similarly, osmolarity, temperature and even digital devices could impact certain CL parameters, such as thickness, diameter, optical zone and wettability [10,12].

The increase in water content and the combination of surface treatments by the SCL industry has led to an advance in wettability, fulfilling the purpose of reducing contact angle hysteresis, generating greater comfort patterns in CL daily use [13]. The inclusion of moisturizers in the CL matrix or surface suggests an increase in tear film volume and stability [14,15,16], playing a vital role in comfort [11], which is not the only clinically significant factor [17]. The lacrimal film promotes corneal function, lubricating and protecting the ocular surface [18]. Inserting a contact lens causes an alteration in the tear film dynamics and divides it into two interfaces, the outermost or pre-lens phase and the post-lens internal phase [19]. The study of tear film with different noninvasive techniques, such as tear rupture times (NIBUT) [20], dehydration of pre-SCL film directly carried in the eye (NIDUT) [10], lipid layer interferometry color pattern [21] and tear meniscus height measurement [22], supposes a useful guide to predict changes in tear stability and is a factor of certainty about the success of the adaptation of CLs. Tear film stability is a good indicator of healthy eye function [23]. The newest development thus far is the water gradient SH-SCL, whose dual structure features a 33% water core and continues to progress to the outside with more surface structure of approximately 80% water, presumably designed to improve use tolerance and minimize the problems associated with SH-SCL [24].

The purpose of this study was to evaluate pre-lens tear film volume, stability and lipid interferometry patterns with a silicone hydrogel water content contact lens through a novel, noninvasive, ocular-surface-analyzer technology.

## 2. Materials and Methods

### 2.1. Design

This longitudinal, single-center prospective study was conducted at the optometry cabinets in the Pharmacy School of the University of Seville. This research was performed according to the Helsinki Declaration and the Ethical Committee Board of the University of Seville (0384-N-22).

### 2.2. Subjects

All subjects included in the study read and signed the informed consent form. An informative sheet was provided to all subjects with the detailed study procedure. The inclusion criteria were as follows: (1) healthy subjects without any eye disease or eye treatment, (2) age between 18 and 35 years old, (3) Contact Lens Dry Eye Questionnaire 8 (CLDEQ8) under 12 score points [25], (4) daily or monthly replacement silicone hydrogel contact lens wearers, (5) manifest objective and subjective spherical equivalent refraction ≤ 4.50 diopters, and (6) manifest objective and subjective refractive astigmatism ≤ 1.00 diopter. The exclusion criteria were as follows: (1) ocular infection or inflammation, with no previous history of ocular surgery, (2) taking any ophthalmic or systemic medications with tear film or ocular surface effects, and (3) pregnancy or breastfeeding.

### 2.3. Materials

Noninvasive analysis of the tear film was assessed with the Integrated Clinical Platform (ICP) Ocular Surface Analyzer (OSA) from SBM System^®^ (Orbassano, Torino, Italy). Detailed information of the device was described in previous research [26]. Meibomian gland evaluation was assessed with the nonmydriatic infrared meibography digital fundus camera Cobra^®^ HD (Construzione Strumenti Oftalmici CSO^®^, Firenze, Italy). The degree of meibomian gland dysfunction (MGD) was measured by the ImageJ method defined by Pult and Nichols [27]. MGD was classified into one of four grades according to the severity of the loss.

Tear volume was measured with Schirmer strips (Tear Flo, HUB Pharmaceutical, Michigan, USA). Two subjective dry eye disease questionnaires were used: the Contact Lens Dry Eye Questionnaire 8 (CLDEQ-8) [25] and the Standard Patient Evaluation of Eye Dryness (SPEED) [28] test.

Regarding the contact lens studied, silicone hydrogel (TOTAL 30^®^, Alcon Inc., Fort Worth, Texas, USA) was used. The Food and Drug Administration (FDA) material group has a high water content and is nonionic (V-B). This contact lens has biomimetic CELLIGENT^®^ Technology that supports resistance to bacteria and lipid deposits. Furthermore, other features were the water gradient technology within a high water content (>90%) at the outermost surface. The technical parameters are presented in Table 1. The contact lens care system solution was a multipurpose solution (MPS) containing 0.00015% polyhexamethylene biguanide (PHMB), 0.01% ethylenediaminetetraacetic acid (EDTA), sodium hyaluronate and hydroxyethyl cellulose in an isotonic, buffered aqueous solution (Lens 55^®^ Care Hyaluropolimer Plus 360 mL, Servilens Fit and Cover^®^, Granada, Spain) for all subjects. Lehfilcon A silicone hydrogel technical parameters are presented in Table 1.

### 2.4. Examination Procedure

In the first phase, subjects were classified according to inclusion and exclusion criteria. The subject’s sample was obtained from the non-optometry academic community. Standard contact lens protocol adaptation was performed according to the Graeme Young Soft Lens Design and Fitting chapter in the Nathan Efron *Contact Lens Practice* book [29]. All subjects were trained to prevent using any lubricants or contact lenses seven days prior to the study. After this wash-out period was finished, subjective questionnaires and noninvasive examination with OSA and meibography was performed [26]. Conjunctival redness classification, lipid layer thickness (LLT), tear meniscus height (TMH), first NIBUT (FNIBUT), mean NIBUT (MNIBUT) and lid opening time (LOT) were included in the protocol.

In a second phase, the subjects were reassessed after 30 min of contact lens wearing to quantify the volume, stability and lipid pattern of the short-term pre-lens tear film. The temperature and humidity area assessment conditions were constant during all measurements. Ocular surface tests were taken alternating between both eyes. Furthermore, between OSA measurement steps, the subjects blinked normally within one minute, and prior to the next measurement, the subject deliberately blinked three full times.

### 2.5. Statistical Analysis

Statistical analysis was performed with SPSS statistical software (version 26.0, IBM Corp, Armonk, NY, USA). Descriptive analysis was performed with the mean ± SD (range value). The normality distribution of the data was assessed with the Shapiro–Wilk test. Differences in qualitative variables were assessed with the chi-squared test. The differences between the previous and short-term pre-lens variables were performed with the Wilcoxon test. The correlation study was evaluated with the Spearman’s rho test. For all tests, the significance level was established at 95% (*p* value < 0.05). The sample size was evaluated with the GRANMO^®^ calculator (Institut Municipal d’Investigació Mèdica, Barcelona, Spain. Version 7.12). The two-sided test was used. The risk of alpha and beta was set at 5% and 20%, respectively. The estimated standard deviation (SD) of the differences was set at 0.45 (based on Marx et al. [30] SD main variable research), the expected minimum pre-lens NIBUT difference was set at 0.30 s, and finally, the loss to follow-up rate was set at 0.00. This achieved a recommended sample size of twenty subjects.

## 3. Results

Sixty-two silicone hydrogel contact fittings were performed in a sample of thirty-one myopic with low astigmatism subjects. Descriptive analyses of sex, nationality, age, noncycloplegic manifest refraction, LogMAR and decimal visual acuity, corneal meridian, contact lens power, Schirmer test, CLDEQ-8 questionnaire, SPEED questionnaire, and superior and inferior eyelid meibomian gland dysfunction are presented in Table 2. Longitudinal ocular surface measurements are presented in Table 3.

**Figure 1:**
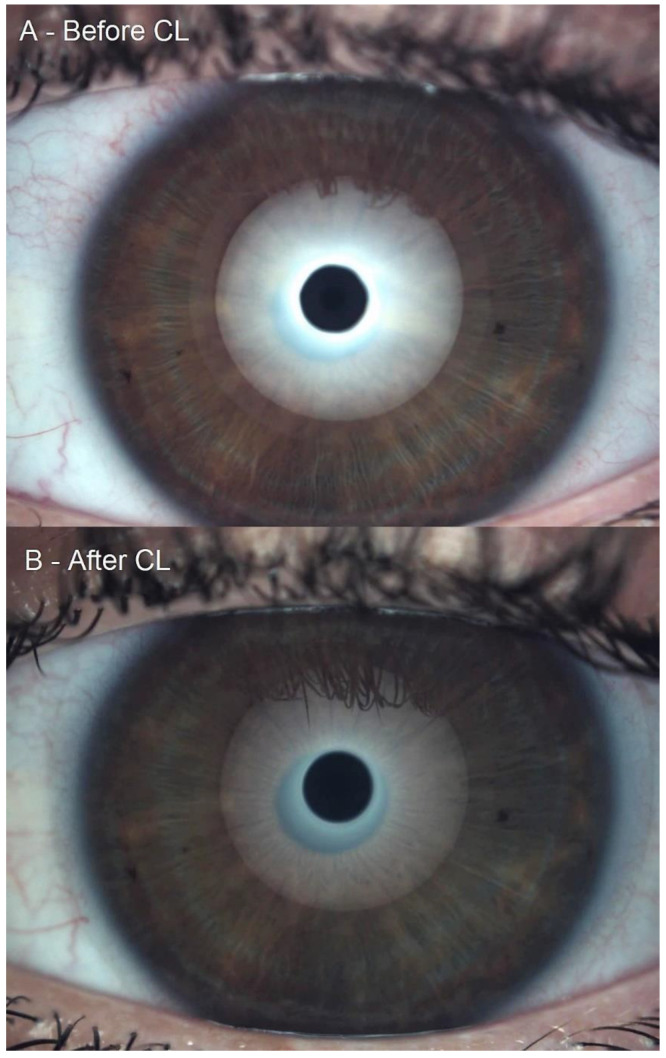
Lipid layer thickness interferometry decreased from grade 2 to grade 0. (**A**): Grade 2 Guillon pattern and (**B**): Grade 0 Guillon pattern.

Conjunctival redness classification achieved a non-statistically significant increase of 0.06 ± 0.30 grades on the Efron Scale (W = 17.50, *p* = 0.10). Conjunctival redness decreased, increased and did not change in 5, 1 and 56 eyes, respectively. A trivial effect size of 0.11 was reported. The rho of Spearman between the previous and posterior conjunctival redness classifications was 0.89 (*p* < 0.01). Lipid layer thickness interferometry decreased 0.14 ± 1.00 grades on the Guillon scale (W = 311.00, *p* = 0.23). Lipid thickness decreased, increased and did not change in 22, 17 and 23 eyes, respectively. A trivial effect size of 0.09 was reported. The rho of Spearman between the previous and posterior conjunctival redness classifications was 0.72 (*p* < 0.01). Lipid layer thickness interferometry decreased from grade 2 to grade 0, as presented in Figure 1. The tear meniscus height remained remarkably similar, with a change of 0.001 ± 0.03 mm (W = 695.00, *p* = 0.76). TMH decreased, increased and did not change in 31, 20 and 11 eyes, respectively. A trivial effect size of 0.01 was reported. The Spearman’s rho between the previous and posterior TMH was 0.79 (*p* < 0.01).

**Figure 2:**
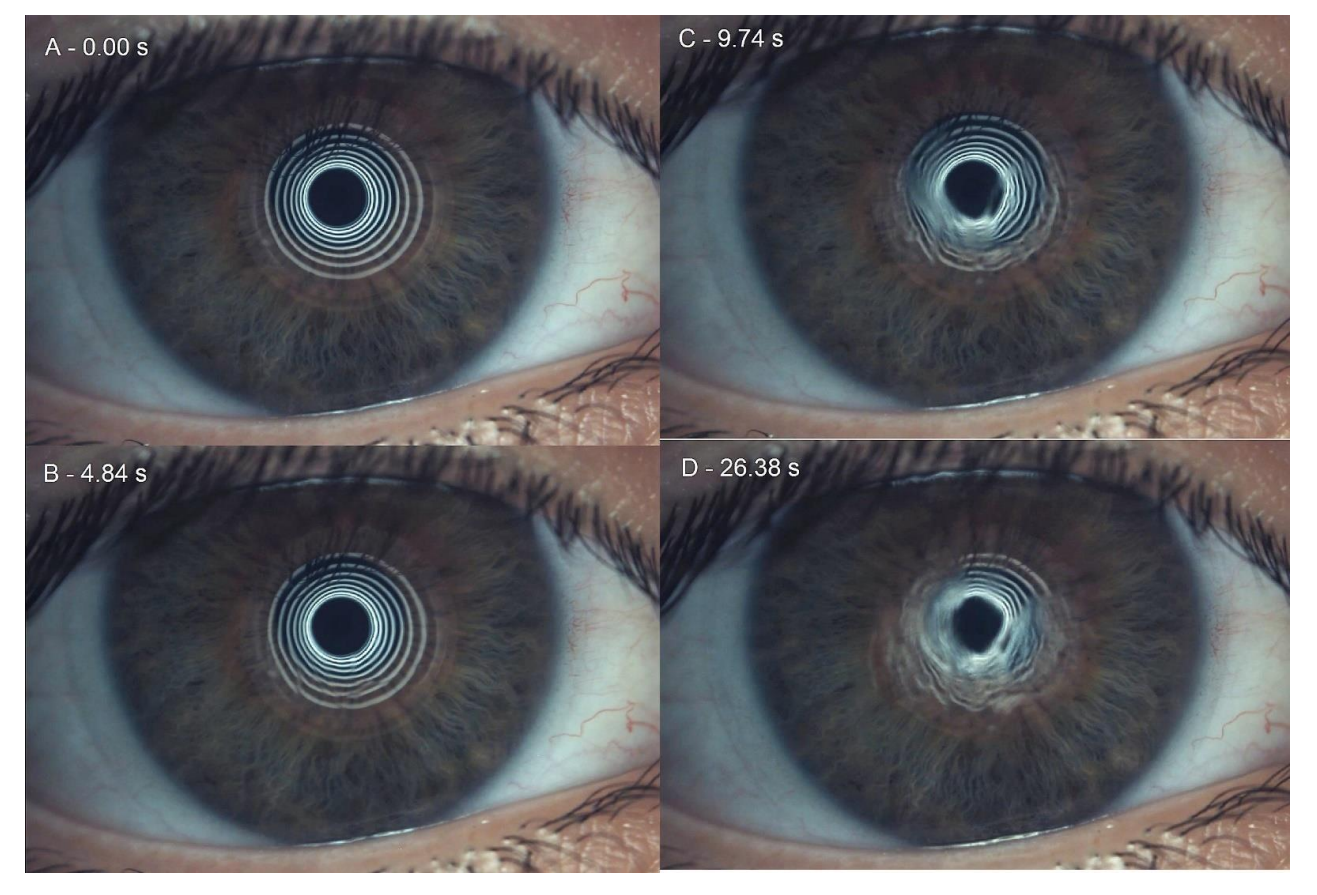
Sequentially captures examples of noninvasive break-up time. (**A**): initial moment, (**B**): first noninvasive break-up time, (**C**): mean noninvasive break-up time and (**D**): last capture prior to lid opening time.

FNIBUT reported a slight decrease of 0.40 ± 1.40 s (W = 600.00, *p* = 0.01). FNIBUT decreased, increased and did not change in 41, 20 and 41 eyes, respectively. A 0.41 moderate effect size was reported. The rho of Spearman between the previous and posterior FNIBUT was 0.00 (*p* = 0.95). A previous FNIBUT achieved a nonsignificant correlation of 0.008 (*p* = 0.95) with the 20-min FNIBUT. MNIBUT achieved a statistically and clinically significant difference of 6.08 ± 10.40 s. (W = 1156.50, *p* < 0.01). MNIBUT decreased and increased in 17 and 45 eyes, respectively. A 0.56 large effect size was reported. The rho of Spearman between the previous and posterior MNIBUT was 0.57 (*p* < 0.01). A previous MNIBUT achieved a significant correlation of 0.57 (*p* < 0.01). Finally, LOT showed the largest increase of 12.21 ± 19.24 s. (W = 1551.50, *p* < 0.01). LOT decreased, increased and did not change in 18, 43 and 1 eyes, respectively. A 0.58 large effect size was reported. The Spearman’s rho between the previous and posterior LOTs was 0.55 (*p* < 0.01). Differences between baseline and short-term results with Lehfilcon A is presented in Table 3. Sequentially captured examples of the initial moment, FNIBUT, MNIBUT and LOT are presented in Figure 2.

## 4. Discussion

In this study, the tear film volume, stability and lipid pattern changes within the pre-lens tear film were assessed in a Lehfilcon A silicone hydrogel contact lens with water gradient technology. Moreover, a novel, noninvasive, ocular-surface-analyzer technology was used. The conjunctival redness classification achieved a non-statistically significant increased percentage. Lipid layer thickness interferometry decreased, and tear meniscus height remained remarkably similar to baseline. However, significant statistical and clinical changes were achieved in FNIBUT and MNIBUT that decreased and increased, respectively. Finally, the LOT increased significantly with Lehfilcon A.

These results are similar to those found by Llorens-Quintana et al. [31], who describe how the FNIBUT decreases with CL use time, without finding a relationship between it and the precorneal NIBUT. It also concludes that the changes in the pre-lens NIBUT would be related to the CL material and not only to the quality of the baseline chronic tear film. In a similar line of research, Montani et al. [32] linked these changes to the CL material. The lens with the highest water content and lowest DK (hydrogel) of those studied had less impact on the tear film characteristics, with fewer changes in TMH and pre-lens NIBUT than other lenses assessed with lower water content and higher DK. However, the high water content suggested that the high water gradient contributes to a lower impact on the wearer’s tear film, as do other surface treatments [33]. The increase in water content and the combination of surface treatments by CL manufacturers has led to an advance in wettability, generating greater comfort patterns in CL daily use [3,11]. The pre-lens tear film stability could change depending on the wettability of the CL material [34].

The decrease in FNIBUT would be related to the decrease in LLT, which, although it does not present significant changes, has a value lower than that measured before putting in the CL, and that would make the tear evaporate faster. However, the Lehfilcon A aqueous gradient would explain the significant increase in MNIBUT and LOT. Fujimoto et al. [24] reported that daily disposable Lehfilcon A contact lenses increase NIBUT and reduce TMH. The results achieved in our study demonstrated that TMH remains stable in a short-term period, so the integrity of ocular physiology could not vary [35]. Several studies have described the reliability of the pre-lens NIBUT measurement [30,34,36] and the importance of tear film stability to guarantee CL comfort, which, as described by Guillon et al. [37], is lower in patients who present symptoms with CLs than in those who do not. Furthermore, the use of interferometry would be more reliable when obtaining this measurement compared to other methods [34]. Finally, Muhafiz and Demir [38] considered that precorneal NIBUT measurements may be useful for diagnosing tear instability, but that pre-lental NIBUT values are not yet capable of adequately defining tear film dynamics in CL users. We consider that this measurement provides a great deal of information on the relationship between the CL and the tear film and that it can help contrast signs and symptoms.

With respect to limitations, more studies would be necessary to establish a relationship between changes in the lipid layer thickness and the decrease in FNIBUT, as well as the increase in the MNIBUT and LOT. Future research should include the influence of unconventional materials and surface treatment on these parameters to help us choose the appropriate CL for each case, especially in users who already have problems with the tear film.

## 5. Conclusions

Silicone hydrogel contact lenses with water gradient technology significantly increased the mean pre-lens NIBUT and lid opening time. Lehfilcon A suggested an improvement in contact lens wearers with tear film instability or decreased subjective symptoms of dry eye disease.

## Figures and Tables

**Table 1:** Lehfilcon A silicone hydrogel technical parameters.

Material	Lehfilcon A
Base Curve	8.4 mm
Diameter	14.2 mm
FDA Group	V-B
Wetting Agent	Phosphoryl Choline
Material/Water (%)	45/55
Center Thickness	0.08 mm
Oxygen Transmission	154 Dk/t
Modulus	0.6 MPa
UV Blocking	Class 1
UVA Blocking	>90%
UVB Blocking	>99%
Light Filter	HEVL
Dynamic Light	No absorption

HEVL: High Energy Visible Light.

**Table 2:** Descriptive analysis of the sample.

Variable	Value
Gender (%)	
Male	14 (22.6)
Female	48 (77.4)
Nationality (%)	
Italian	21 (67.75)
Spanish	4 (12.90)
Mexican	2 (6.46)
Slovak	1 (3.22)
Polish	1 (3.22)
Germany	1 (3.22)
Austrian	1 (3.22)
Age (Years)	22.23 ± 1.39 (19 to 25)
Sphere (Diopters)	−2.64 ± 1.15 (−5.50 to −0.50)
Cylinder (Diopters)	−0.44 ± 0.37 (−1.50 to 0.00)
Axis (Degrees, °)	111.44 ± 70.08 (5.00 to 180.00)
Visual Acuity (Log MAR)	−0.03 ± 0.05 (−0.10 to 0.10)
Visual Acuity (Decimal)	1.07 ± 0.10 (0.80 to 1.20)
Flat Corneal Meridian (mm)	7.87 ± 0.31 (7.40 to 8.74)
Steep Corneal Meridian (mm)	7.73 ± 0.29 (7.25 to 8.61)
Mean Corneal Meridian (mm)	7.80 ± 0.30 (7.37 to 8.67)
Contact Lens Power (Diopters)	−2.56 ± 1.12 (−5.00 to −0.75)
Schirmer Test (mm)	30.21 ± 8.43 (6.00 to 35.00)
CLDEQ8 (Score Points)	11.32 ± 5.56 (1.00 to 29.00)
SPEED Test (Score Points)	7.39 ± 4.39 (0.00 to 15.00)
Superior Eyelid MGD (%)	28.87 ± 15.11 (10.30 to 96.20)
Inferior Eyelid MGD (%)	49.69 ± 17.86 (17.00 to 87.30)

CLDEQ8: Contact Lens Dry Eye Questionnaire, SPEED: Standard Patient Evaluation of Eye Dryness.

**Table 3:** Ocular surface longitudinal changes before and with silicone hydrogel wearing.

Variable	Before Lehfilcon A	30-min with Lehfilcon A	*p* Value
Conjunctival Redness Classification (Efron Scale)	1.08 ± 0.63 (0.00 to 2.00)	1.15 ± 0.56 (0.00 to 2.00)	0.10
Lipid Layer Thickness Interferometry (Guillon Pattern)	2.05 ± 1.53 (0.00 to 5.00)	1.90 ± 1.73 (0.00 to 5.00)	0.23
Tear Meniscus Height (Millimeters)	0.21 ± 0.04 (0.11 to 0.32)	0.21 ± 0.06 (0.07 to 0.32)	0.76
First NIBUT (Seconds)	5.03 ± 1.04 (3.60 to 7.80)	4.63 ± 0.89 (3.64 to 8.52)	0.01 *
Mean NIBUT (Seconds)	15.19 ± 9.54 (4.50 to 49.76)	21.27 ± 11.97 (5.44 to 56.48)	<0.01 *
Lid Opening Time (Seconds)	26.36 ± 19.72 (5.04 to 93.60)	38.58 ± 21.78 (7.04 to 107.04)	<0.01 *

NIBUT: Non-Invasive Break Up Time. * Statistically significant within Wilcoxon test.

## Data Availability

The data presented in this study are available on request from the corresponding author. The data are not publicly available due to their containing information that could compromise the privacy of research participants.

## Abbreviations

CL	Contact Lens
CLDEQ8	Contact Lens Dry Eye Questionnaire 8
EDTA	Ethylenediaminetetraacetic Acid
FDA	Food and Drug Administration
FNIBUT	First Non-Invasive Break-Up Time
ICP	Integrated Clinical Platform
LLT	Lipid Layer Thickness
LOT	Lid Opening Time
MGD	Meibomian Gland Dysfunction
MNIBUT	Mean Non-Invasive Break-Up Time
NIBUT	Non-Invasive Break-Up Time
NIDUT	Non-Invasive Dehydration-Up Time
OSA	Ocular Surface Analyzer
SCL	Soft Contact Lens
SH-SCL	Silicone Hydrogel Soft Contact Lens
SPEED	Standard Patient Evaluation of Eye Dryness
TMH	Tear Meniscus Height

## References

[1] Tahhan N., Naduvilath T.J., Woods C., Papas E. (2022). Review of 20 years of soft contact lens wearer ocular physiology data. Contact Lens Anterior Eye.

[2] Jacob J.T. (2013). Biocompatibility in the development of silicone-hydrogel lenses. Eye Contact Lens.

[3] Eftimov P.B., Yokoi N., Peev N., Paunski Y., Georgiev G.A. (2021). Relationships between the material properties of silicone hydrogels: Desiccation, wettability and lubricity. J. Biomater. Appl..

[4] Tauste A., Ronda E., Baste V., Bråtveit M., Moen B.E., Seguí Crespo M. (2018). del M. Ocular surface and tear film status among contact lens wearers and non-wearers who use VDT at work: Comparing three different lens types. Int. Arch. Occup. Environ. Health.

[5] Musgrave C.S.A., Fang F. (2019). Contact lens materials: A materials science perspective. Materials.

[6] Lira M., Silva R. (2017). Effect of Lens Care Systems on Silicone Hydrogel Contact Lens Hydrophobicity. Eye Contact Lens.

[7] Markoulli M., Kolanu S. (2017). Contact lens wear and dry eyes: Challenges and solutions. Clin. Optom..

[8] McMonnies C.W. (2021). Could contact lens dryness discomfort symptoms sometimes have a neuropathic basis?. Eye Vis..

[9] Rex J., Knowles T., Zhao X., Lemp J., Maissa C., Perry S.S. (2018). Elemental Composition at Silicone Hydrogel Contact Lens Surfaces. Eye Contact Lens.

[10] Guillon M., Patel T., Patel K., Gupta R., Maissa C.A. (2019). Quantification of contact lens wettability after prolonged visual device use under low humidity conditions. Contact Lens Anterior Eye.

[11] Eftimov P., Yokoi N., Peev N., Georgiev G.A. (2019). Impact of air exposure time on the water contact angles of daily disposable silicone hydrogels. Int. J. Mol. Sci..

[12] Lee S.E., Kim S.R., Park M. (2015). Oxygen permeability of soft contact lenses in different pH, osmolality and buffering solution. Int. J. Ophthalmol..

[13] Tighe B.J. (2013). A decade of silicone hydrogel development: Surface properties, mechanical properties, and ocular compatibility. Eye Contact Lens.

[14] Maulvi F.A., Patel P.J., Soni P.D., Desai A.R., Desai D.T., Shukla M.R., Ranch K.M., Shah S.A., Shah D.O. (2020). Novel Poly(vinylpyrrolidone)-Coated Silicone Contact Lenses to Improve Tear Volume during Lens Wear: In Vitro and in Vivo Studies. ACS Omega.

[15] Singh A., Li P., Beachley V., McDonnell P., Elisseeff J.H. (2015). A hyaluronic acid-binding contact lens with enhanced water retention. Contact Lens Anterior Eye.

[16] Chang W.H., Liu P.Y., Lin M.H., Lu C.J., Chou H.Y., Nian C.Y., Jiang Y.T., Hsu Y.H.H. (2021). Applications of hyaluronic acid in ophthalmology and contact lenses. Molecules.

[17] García-Montero M., Rico-del-Viejo L., Llorens-Quintana C., Lorente-Velázquez A., Hernández-Verdejo J.L., Madrid-Costa D. (2019). Randomized crossover trial of silicone hydrogel contact lenses. Contact Lens Anterior Eye.

[18] Bai Y., Nichols J.J. (2017). Advances in thickness measurements and dynamic visualization of the tear film using non-invasive optical approaches. Prog. Retin. Eye Res..

[19] Muntz A., Subbaraman L.N., Sorbara L., Jones L. (2015). Tear exchange and contact lenses: A review. J. Optom..

[20] Graham A.D., Lin M.C. (2021). The relationship of pre-corneal to pre-contact lens non-invasive tear breakup time. PLoS ONE.

[21] Bai Y., Ngo W., Nichols J.J. (2019). Characterization of the thickness of the tear film lipid layer using high resolution microscopy. Ocul. Surf..

[22] Binotti W.W., Bayraktutar B., Ozmen M.C., Cox S.M., Hamrah P. (2020). A Review of Imaging Biomarkers of the Ocular Surface. Eye Contact Lens.

[23] Willcox M.D.P., Argüeso P., Georgiev G.A., Holopainen J.M., Laurie G.W., Millar T.J., Papas E.B., Rolland J.P., Schmidt T.A., Stahl U. (2017). TFOS DEWS II Tear Film Report. Ocul. Surf..

[24] Fujimoto H., Ochi S., Yamashita T., Inoue Y., Kiryu J. (2021). Role of the water gradient structure in inhibiting thin aqueous layer break in silicone hydrogel-soft contact lens. Transl. Vis. Sci. Technol..

[25] Chalmers R.L., Keay L., Hickson-Curran S.B., Gleason W.J. (2016). Cutoff score and responsiveness of the 8-item Contact Lens Dry Eye Questionnaire (CLDEQ-8) in a Large daily disposable contact lens registry. Contact Lens Anterior Eye.

[26] Sánchez-González M.C., Capote-Puente R., García-Romera M.-C., De-Hita-Cantalejo C., Bautista-Llamas M.-J., Silva-Viguera C., Sánchez-González J.-M. (2022). Dry eye disease and tear film assessment through a novel non-invasive ocular surface analyzer: The OSA protocol. Front. Med..

[27] Pult H., Nichols J.J. (2012). A review of meibography. Optom. Vis. Sci..

[28] Baudouin C., Aragona P., Van Setten G., Rolando M., Irkeç M., Del Castillo J.B., Geerling G., Labetoulle M., Bonini S. (2014). Diagnosing the severity of dry eye: A clear and practical algorithm. Br. J. Ophthalmol..

[29] Young G., Efron N. (2018). Soft Lens Design and Fitting. Contact Lens Practice.

[30] Marx S., Eckstein J., Sickenberger W. (2020). Objective analysis of pre-lens tear film stability of daily disposable contact lenses using ring mire projection. Clin. Optom..

[31] Llorens-Quintana C., Mousavi M., Szczesna-Iskander D., Iskander D.R. (2018). Non-invasive pre-lens tear film assessment with high-speed videokeratoscopy. Contact Lens Anterior Eye.

[32] Montani G., Martino M. (2020). Tear film characteristics during wear of daily disposable contact lenses. Clin. Ophthalmol..

[33] Vidal-Rohr M., Wolffsohn J.S., Davies L.N., Cerviño A. (2018). Effect of contact lens surface properties on comfort, tear stability and ocular physiology. Contact Lens Anterior Eye.

[34] Itokawa T., Suzuki T., Iwashita H., Hori Y. (2020). Comparison and evaluation of prelens tear film stability by different noninvasive in vivo methods. Clin. Ophthalmol..

[35] Müller C., Marx S., Wittekind J., Sickenberger W. (2020). Subjective comparison of pre-lens tear film stability of daily disposable contact lenses using ring mire projection. Clin. Optom..

[36] Marx S., Sickenberger W. (2017). A novel in-vitro method for assessing contact lens surface dewetting: Non-invasive keratograph dry-up time (NIK-DUT). Contact Lens Anterior Eye.

[37] Guillon M., Dumbleton K.A., Theodoratos P., Wong S., Patel K., Banks G., Patel T. (2016). Association between contact lens discomfort and pre-lens tear film kinetics. Optom. Vis. Sci..

[38] Muhafiz E., Demir M.S. (2022). Ability of non-invasive tear break-up time to determine tear instability in contact lens wearers. Int. Ophthalmol..

